# Iron accumulation and lipid peroxidation: implication of ferroptosis in diabetic cardiomyopathy

**DOI:** 10.1186/s13098-023-01135-5

**Published:** 2023-07-19

**Authors:** Xuehua Yan, Yang Xie, Hongbing Liu, Meng Huang, Zhen Yang, Dongqing An, Guangjian Jiang

**Affiliations:** 1 College of Traditional Chinese Medicine, Xinjiang Medical University, Xinjiang, China; 2 Xinjiang Key Laboratory of Famous Prescription and Science of Formulas, Xinjiang, China; 3 Affiliated Hospital of Traditional Chinese Medicine of Xinjiang Medical University, Xinjiang, China

**Keywords:** Ferroptosis, Lipid peroxidation, Diabetic cardiomyopathy, Diabetes

## Abstract

Diabetic cardiomyopathy (DC) is a serious heart disease caused by diabetes. It is unrelated to hypertension and coronary artery disease and can lead to heart insufficiency, heart failure and even death. Currently, the pathogenesis of DC is unclear, and clinical intervention is mainly symptomatic therapy and lacks effective intervention objectives. Iron overdose mediated cell death, also known as ferroptosis, is widely present in the physiological and pathological processes of diabetes and DC. Iron is a key trace element in the human body, regulating the metabolism of glucose and lipids, oxidative stress and inflammation, and other biological processes. Excessive iron accumulation can lead to the imbalance of the antioxidant system in DC and activate and aggravate pathological processes such as excessive autophagy and mitochondrial dysfunction, resulting in a chain reaction and accelerating myocardial and microvascular damage. In-depth understanding of the regulating mechanisms of iron metabolism and ferroptosis in cardiovascular vessels can help improve DC management. Therefore, in this review, we summarize the relationship between ferroptosis and the pathogenesis of DC, as well as potential intervention targets, and discuss and analyze the limitations and future development prospects of these targets.

## Introduction

Diabetic cardiomyopathy (DC) is a common diabetic cardiovascular complication of ventricular insufficiency in diabetic patients in the absence of coronary atherosclerosis and hypertension [1]. DC can occur in both type 1 and type 2 diabetes mellitus [2]. The occurrence and development of DC are associated with hyperglycemia [3], insulin resistance [4], mitochondrial dysfunction [5, 6], reactive oxygen species (ROS) accumulation [7], microvascular dysfunction [8] and other factors are closely related. So far, although the above factors have provided the direction for the pathogenesis of DC, the exact pathogenesis of DC is still unclear. In addition to strict control of blood glucose [9], using the traditional and novel hypoglycemic drugs [10, 11], there is still a lack of intervention for the REDOX imbalance caused by the disorder of glucose and lipid metabolism and iron metabolism, at present, and the targeted intervention for the pathogenesis of DC needs further research.

Recent studies have shown that ferroptosis is essential in the pathogenesis of DC [12–15]. Iron overload may promote the introduction of polyunsaturated fatty acids into the cell membrane, leading to an excessive accumulation of lipid hydroperoxides and an imbalance of REDOX [16, 17], thus mediating regulatory cell death, i.e. ferroptosis. Wang et al. [12] confirmed the real existence of ferroptosis in cardiomyocytes of DC and its damaging effect on the heart by in vivo and in vitro experiments, respectively. In the in vitro experiment, the expression of lipid peroxidation marker malondialdehyde and ferroptosis marker Ptgs2 in DC cardiomyocytes was increased, and the expression of SLC7A11, glutathione (GSH) level and ferritin level were decreased. Moreover, the typical ferroptosis morphological changes of DC mouse cardiomyocytes were confirmed by transmission electron microscopy [12], which further confirmed the destructive effect of ferroptosis on the structure and function of cardiomyocytes, and emphasized the important significance of ferroptosis in the myocardial injury of DC [12, 18].

Early studies have found that myocardial apoptosis expressed in diabetic tissues is 85 times that in non-diabetic tissues [19, 20]. Jinjing et al. [21] pointed out that there are various forms of cell death in DC, and inhibiting any form of cardiac cell death will have a huge protective effect on DC. A number of experiments have confirmed this view, namely, inhibiting ferroptosis is beneficial to reducing myocardial injury [22–24], effectively preventing cardiomyocytes and improve cardiac dysfunction [12, 24], and delaying the progression of DC. Inhibiting ferroptosis provides a new research direction for clinical intervention of DC. Applying iron chelating agents reduced the level of oxidation stress, inflammation, and myocardial remodeling [25, 26]. However, the current research on iron death in DC is still limited to animal experiments and cell experiments, and the research and understanding of human beings are still very limited.

Therefore, we summarized iron metabolism, lipid metabolism and ferroptosis related signaling pathways, analyzed and discussed the related cellular and molecular mechanisms of ferroptosis in myocardial injury and microvascular injury of DC. The potential intervention targets and future research prospects of ferroptosis in DC are comprehensively discussed, which provides a new research direction for the clinical diagnosis and treatment of DC.

## Molecular mechanism of ferroptosis

**Fig. 1:**
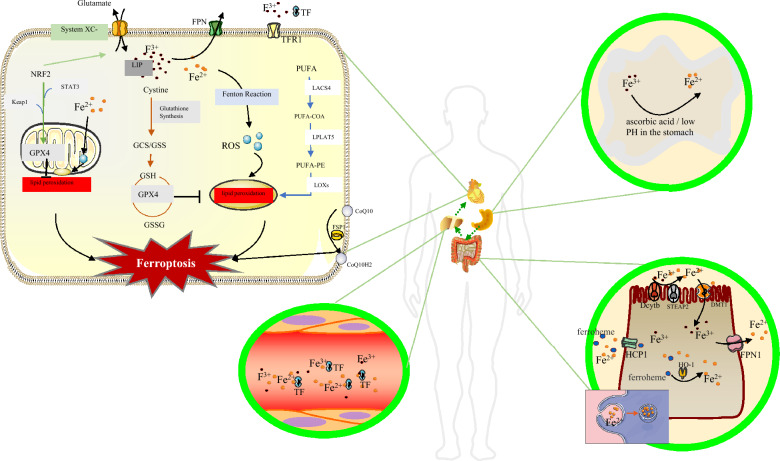
Dietary iron is absorbed by the gastrointestinal tract [55–57], metabolized by the liver into the circulatory system, and distributed to cardiomyocytes on demand [61]. Ferroptosis is closely related to iron metabolism, amino acid metabolism, lipid metabolism and other metabolic pathways

Unlike other programmed apoptosis mechanisms, ferroptosis is specific in genetics, morphology, immunology, and biochemistry, as shown in Table 1. Ferroptosis is closely related to iron metabolism, amino acid metabolism, lipid metabolism and other metabolic pathways, as shown in Fig. 1. Therefore, this part focuses on introducing the pathogenesis of DC mediated by ferroptosis under the action of the above molecular metabolic pathways.

**Table 1:** Type of cell death

Type of cell death	Definition	Feature and regulation		Disease	References
Autophagy	Removes abnormal proteins and organelles and maintains cell homeostasis by removing misfolded proteins and damaged organelles and invading microorganisms through lysosomes	A large vacuole of the cytoplasm, the formation of autophagosomes, phagocytosis and subsequent lysosomal degradation		Infection; immune disease; metabolic disease; cancer; neurodegenerative	[27, 28]
Apoptosis	Citing no inflammatory responses regulated by genes, which plays important roles in maintain cells stability in tissues, immune and defense responses, growth and development of tumors, and cells damage caused by poisoning	The contraction of cytoplasm, shrinkage of the nucleus, rupture of the nucleus, fragmentation of DNA, foaming of plasma membranes, and formation of apoptosis bodies		Cancer; atherosclerosis, diabetes; hepatic fibrosis; wound healing	[29, 30]
Necroptosis	A type of regulated necrosis that critically depends on RIPK3 and MLKL	Organelles and plasma membrane rupture, and the final treated cell cadavers were not obviously involved in phagocytes and lysosomes		Viral infection; acute kidney injury; cardiac ischemia/reperfusion; human tumors	[31]
Ferroptosis	A form of cell death driven by iron-dependent lipid peroxidation	Genetics	Regulated by multiple genes, mainly the changes of iron homeostasis and lipid peroxidation genes	Aging; immunity; cancer; cardiovascular diseases; ischemic-reperfusion	[32, 33]
		Morphology	The mitochondrial volume decreased, the density of double-layer structure increased, the crest decreased or disappeared		
		Immunology	Injury-related molecular pattern molecules release inflammatory mediators to trigger inflammatory response		
		Biochemistry	GSH consumption, GPX4 activity decreased, Fe^2+^ mediated lipid peroxidation through Fenton reaction, produce excess ROS		
Cuproptosis	Copper directly binds to lipoylated components of the tricarboxylic acid (TCA) cycle, causing protein aggregation, iron-sulfur cluster protein loss, proteotoxic stress, and cell death	The key regulators:	FDX1 and protein lipoylation	Wilson’s; Alzheimer's disease; Parkinson's disease	[34–36]
		Function channel:	Mitochondrial respiration		
		Upstream regulator	FDX1		
Pyroptosis	A form of programmed cell death mediated by the N-terminal fragment of gasdermin D that gives rise to inflammation via the release of some proinflammatory cytokines, including IL-1β, IL-18 and HMGB1	The canonical signalling pathway dependent on caspas-1 and the non-canonical signalling pathway determined by caspas-4/5/11		Fibrosis; Kidney disease; atherosclerosis; diabetes; neurodegenerative diseases;	[37–40]

### Iron metabolism

One of the vital trace elements in the human body is iron. On the one hand, as a cofactor, in the form of reduced state (Fe^2+^), iron is widely involved in the synthesis of human DNA, iron-sulfur clusters and hemoglobin [41, 42], regulating the activities of lipoxygenase, mitochondrial complex I and II and other enzymes [43, 44], and regulating the proliferation and differentiation of cells. On the other side, through the Fenton reaction, iron can also result in the creation of poisonous oxygen free radicals (hydroxyl radicals). Therefore, iron metabolism needs to be tightly regulated at the cellular and biological levels. Dietary iron intake can be absorbed into intestinal cells in four ways: part of iron ions are absorbed into cells by heme carrier protein 1 transporter (HCP1) on duodenal intestinal cells [45, 46], or dietary ferritin is absorbed into intestinal cells by some entosis mechanism [47]. Low PH in the stomach or ascorbic acid can decrease the remaining Fe^3+^ bound to citrate to Fe^2+^. In addition, duodenal cytochrome b (Dcytb) and prostatic 6 transmembrane epithelial antigen 2 (STEAP2) proteins on the top membrane of intestinal cells can use ascorbic acid oxidation to reduce Fe^3+^ to Fe^2+^. Reduced Fe^2+^ is transported into intestinal cells via divalent metal transporter 1 (DMT1, also known as Nramp2, SLC11A2, and DCT1) or zinc transporter Zrt-Irt‐like proteins 14 and 8 (ZIP 14/8) [48–50]. Heme can be broken down inside the cell or taken into the bloodstream, and heme oxygenase (HO1) can break down the heme groups to liberate free iron (Fe^2+^). Intracellular iron regulation is strictly dependent on the REDOX state (Fe^2+^/Fe^3+^) and is usually stored as a protein in the oxidized state of Fe^3+^ in the unstable iron cisterna (LIP) [51, 52] or through the cytoplasmic transport of ferritin 1 (FPN1, Also known as MTP1, IREG1 and SLC40A1) are transported to the extracellular blood [53] and transported to the circulatory system by the liver transport protein transferrin (TF) [54].

Several important regulators affect the intracellular iron content and homeostasis. NRF2 is a key regulator of cellular oxidation stability, encoded by the NFE2L2 gene and belonging to the leucine zipper structure family [55]. Genes associated to iron excess and lipid peroxidation are also NRF2 target genes [56]. NRF2 controls the transcription of the iron metabolism-related gene SLC40A1 in the nucleus [57]. NRF2, a crucial transcription factor for preserving oxidative homeostasis, is activated by severe oxidative stress to encourage the transcription of the target genes GPX4, HO-1, and SLC7A11 [58, 59]. By controlling the production of iron metabolism-related proteins, transcription modification after interlinking iron regulatory proteins (IRPs) and iron response elements (IREs) is engaged in the regulation of cellular iron homeostasis [60]. In addition, to further reduce the intracellular iron production of transferrin, ferriophagy can control the nuclear receptor coactivator 4 (NCOA4) intracellular iron regulator. Iron-responsive element-binding protein 2 (IREB2), another important encoder, primarily influences transferrin expression and iron transport [61].

The control of iron homeostasis can be achieved by post-transcriptional regulation in addition to transcriptional regulation. According to a study, FPN1 expression is reduced in NRF2 knockout mice [62]. FPN1 is the only iron-release related protein discovered in mammals, and it is encoded by the SLC40A1 gene [63]. It is important for systemic iron homeostasis. Recent research has revealed that altering NRF2 expression in the nucleus can influence its transcriptional target FPN1, resulting in decreased iron outflow and increased intracellular iron accumulation [64]. During myocardial cell injury, NRF2 is transferred from the cytoplasm to the nucleus, thereby activating the transcription of its target gene FPN1 to limit iron ptosis. Low expression of FPN1 exacerbates iron ptosis and intracellular iron buildup in diabetic heart disease. However, in diabetic rats, activation of NRF2 overexpression can greatly increase the transcription of FPN1 and minimize myocardial damage [65]. Different from the whole-body iron regulation pathway, iron metabolism in cardiomyocytes is mainly through the secretion function of the heart itself to promote the up-regulation of cardiac iron modulin protein to achieve iron homeostasis [66]. It may thus be more evidence that cardiomyocytes are more susceptible to iron overload than other cell types as only the FPN protein is accessible for iron output during iron accumulation.

### System XC^−^/GSH/GPX4 pathway

The fundamental cause of ferroptosis is an unbalanced cellular antioxidant defense system, namely the inactivation of the XC-GSH-GPX4-dependent antioxidant defense system, which leads to the buildup of lipid hydroperoxide. ROS antagonism relies on the biosynthesis of GSH, which is introduced or sulfurized by cystine, and selenium, which is essential for the proper functioning of GPX4. The system XC- consists of two subunits, SLC7A11 and SLC3A2, which are anti-transporter of cystine and glutamate and are responsible for transporting extracellular cystine into the cell and reducing it to cysteine. Cystine can be synthesized de novo from methionine through the sulfur transfer pathway [67], or imported into cells by other transporters such as SLC1A1 as substitutes [68]. Cysteine, glutamic acid and glycine were used as substrates to synthesize glutathione. Because of its limited concentration in cells, cysteine is considered a key rate-limiting step in the synthesis of GSH.

GPX4 is the main scrubber of lipid peroxides in cells, which can directly reduce lipid peroxides in membranes to non-toxic lipid alcohols. The esterification of oxidized fatty acids and cholesterol hydroperoxides can only be reduced by GPX4, a member of the GPX family of enzymes [69]. Selenium is an essential component of selenium-containing cysteine proteins (including but not limited to GPX4), which can boost cell antioxidant capacity during ferritic damage and is required for GPX4's REDOX enzyme function [70, 71]. Selenocysteine can not only reduce selenol (SeH) but also oxidize selenic acid (SeOH) in the reduction process of lipid hydroperoxide catalyzed by GPX4. This dynamic process includes: (1) the selenoalcohol form of selenocysteine (GPX4-SeH) is converted to selenoacid intermediate (GPX4-SeOH) by hydrogen peroxide, and hydrogen peroxide (LOOH) is reduced to alcohol (LOH); (2) The intermediate reacts with glutathione to form water and selenium-glutathione admixture (GPX4-Se-SG); (3) Systemic XC-mediated cystine absorption, followed by GSH synthesis and GPX4 activation, is critical in protecting cells from iron sagging. Therefore, a deficiency of selenium in serum or cytoplasm is likely to impair GPX4 function and ultimately lead to the accumulation of lipid peroxides, leading to iron sagging [72]. Studies have revealed that GSH is a crucial cofactor for GPX4 and that its availability controls its capacity to operate normally. The XC system, which is widely distributed in the phospholipid bilayer, controls GPX4 activity, that’s dependent on the exchange of extracellular cystine and intracellular glutamate.

Recently, it was discovered that FSP1 works independently of glutathione to stop harmful peroxidation by rebuilding reduced coenzyme Q10 [69, 73]. CoQ, also known as ubiquitone, CoQ10 is the most common form of CoQ used as a dietary supplement. CoQ10 is an endogenous inhibitor of iron sagging [74]. By transporting electrons from complex I and II to complex III, CoQ10 plays a crucial part in the mitochondrial electron transport chain. Additionally, ubiquitin alcohol (CoQ10H2), the reduced form of CoQ10, is employed as a powerful lipophilic antioxidant in the recovery of other antioxidants like tocopherol and ascorbate. AIFM2(apoptosis-inducing factor mitochondrial associated 2)/FSP1 has been identified as an inhibitor of iron sagging through CoQ10 production [69, 73], which is parallel to the GSH-dependent GPX4 pathway.

Other metabolic processes for amino acids involved in ferroptosis likewise heavily rely on the meglutarate pathway. It controls the manufacture of selenoproteins and other antioxidant molecules like ubiquitin more than any other cellular metabolic process [75]. Isopentenyl pyrophosphate, a metabolic intermediary of the mevalerate pathway, is necessary for the creation of numerous compounds, including ubiquitin [76]. Ubiquitin alcohol can inhibit lipid peroxidation in plasma membrane and block iron ptosis. The antagonist of FIN56 is ferropendant inhibitory protein 1 (FSP1, formerly known as AIFM2), an enzyme that catalyzes the conversion of ubiquitin-ketone to ubiquitin-alcohol [77]. Although practically all lipid membranes contain ubiquitin ketone, only locations outside of the mitochondria are protected from lipid peroxidation by FSP1 dependent ubiquitin ketone modification [77]. In addition, the dysfunction of the xc-system that leads to glutathione depletion also occurs in ferroptosis [78–80]. Oxidative glutamate intoxication, also known as oxidative glutamate intoxication, is a glutamate-induced cell death mediated by blocking of the xc-system [81].

### Lipid metabolism

The production of lipid peroxides is iron-dependent. A high iron diet increases their susceptibility to iron ptosis by increasing extracellular iron concentration [82, 83]. Heat shock protein B family member 1 (HSPB1) and other proteins that reduce intracellular iron levels can also affect intracellular iron sensitivity [84]. The release of iron from ferritin is mostly dependent on ferritin phagocytosis, which is mediated by nuclear receptor coactivator 4 (NCOA4). When ferritin is phagocytosed, NCOA4 attaches to it and moves it to the lysosomes for iron release and destruction [85]. High intracellular iron concentration may then further induce ferroptosis.

Iron participates in lipid oxidation in the following three possible ways:(I) Fenton reaction [86], in which Fe^2+^ acts as a catalyst to supply electrons to O_2_ or H_2_O_2_, promoting the production of ROS and lipid peroxides; (II) Lipid autooxidation catalyzed by iron-catalyzed enzymes; (III) Lipid peroxidation catalyzed by iron-containing LOX [78]. NRF2 plays a key role in mediating glycolipid toxic-dependent ferric sagging in H9C2 cardiomyocytes [18]. At the molecular level, glucolipid toxicity is most likely caused by activation of NRF2-driven ACSL4 transcription and inhibition of FSP1 expression, inhibition of NRF2-regulated GPX4 transcription and damage of NRF2-coordinated iron metabolism gene network, thus leading to iron death of cardiomyocytes and promoting the progression of DC [18].

In addition to intracellular iron buildup, iron ptosis is also caused by a disturbance of lipid metabolism. Arachidonic acid and docosahexaenoic acid are two polyunsaturated fatty acids (PUFAs) found in phospholipids like phosphatidylcholine that mediate intracellular signal transmission and are most vulnerable to oxidation during ferroptosis [86]. PUFAs will undergo repetitive peroxidation when iron-dependent ROS levels are too high, which will cause cell membrane malfunction and eventually lead to iron-dependent cell death [87]. Free PUFAs are susceptible to lipid peroxidation and substrates for lipid signal transduction production. ACSL4 [87] and LPCAT3 [88] exerts stringent control over the peroxide substrate PUFAs at the unsaturated level. The amount of intracellular lipid peroxidation and, consequently, the outcome of ferroptosis are determined by the abundance and distribution of PUFA. The three main characteristics of ferroptosis and associated indications of diabetes mellitus and its consequences are the down-regulation of GPX4 expression, clearance of lipid reactive oxygen species, and rise in mRNA level [89–91].

Some genes that control PUFA production and preserve the integrity of healthy cell membranes may also have an impact on the emergence of ferroptosis in addition to lipid peroxidase. The most significant indicator of ferroptosis is arachidonoyl-(AA-) OOH-phosphatidylethanolamine (PE) [92]. The conversion of AA to AA-COA is driven by the long chain family of acyl-CoA synthases 4 (ACSL4). ACSL4, a member of the long chain family of acyl-CoA synthases, is a reliable marker of ferroptosis and can induce the production of 5-hydroxylethyldieneacetic acid (5-HETE), the signature signal of ferroptosis [93]. Unsaturated arachidonic acid is demonstrated to be inserted into phosphatidylethanolamine in the phospholipid membrane of cells by lysophosphatidyl choline acyltransferase 3 (LPCAT3) and ACSL4, later, AA-coa is converted to AA-PE by esterification promoted by LPCAT3 [87, 93]. In the last step, the formation of AA-oh-PE requires the oxidation of AA-PE catalyzed by lipoxyphenase (LOXs) [94]. Ultimately, uncontrolled accumulation of AA-oh-PE leads to iron sagging and exacerbates erastin and RSL3-induced ferroptosis in cancer cells [94].

## The cellular processes of ferroptosis in DC

In the early phases of the condition, DC typically causes cell death, myocardial fibrosis, left ventricular hypertension, unfavorable remodeling, and progression of diastolic dysfunction [95, 96]. At this stage, if strict metabolic control is applied, DC pathological changes can be reversed. With the progression of the disease, DC deforms to systolic dysfunction with reduced ejection fraction (EF) in the advanced stage, and its myocardial changes are irreversible, resulting in the final clinical manifestations of heart failure [97, 98]. Excessive oxidative stress mediated by lipid peroxidation and iron overload is one of the important causes of myocardial injury in DC. Many studies have demonstrated that diabetic heart disease can be effectively treated by targeting the source of oxidative stress or endogenous antioxidant defense systems [99–102], as well as removing ROS [103]. Therefore, inhibiting lipid peroxidation caused by ferroptosis and iron overload may be of great significance to enhance the antioxidant capacity of the body and reduce the pathological damage of DC [100]. This section focuses on the role of ferroptosis in DC pathological changes and related pathways, and Table 2 summarizes the role of iron-related protein in heart metabolism confirmed by recent experiments.

**Table 2:** Iron metabolism disturbances described in heart

Iron metabolism level	Protein	Experimental procedure	Result	References
Iron absorption	HO‐1	6 months of myricetin treatment (200 mg/kg/d) in DC mice	IκBα/NFκB inhibited and Nrf2/HO-1 enhanced	[104]
			Cardiac hypertrophy, apoptosis, and interstitial fibrosis improved	
	Dcytb	In MDCK cells expressing an inducible duodenal cytochrome b-green fluorescent protein fusion construct	DMT1 and copper transporter 1 help Dcytb ingest iron and copper	[105]
	Hephaestatin	The whole body and intestine-specific hephaestin knockout mice	Hephaestin is not necessary because both mouse strains survived	[106]
			Duodenal enterocytes stored iron in knockout mice, reducing intestinal iron absorption	
	TFR1	Used wild-type and autophagy-deficient cells, BECN1^±^ and LC3B^−/−^	During ferroptosis, ROS-induced autophagy controls ferritin degradation and TfR1 expression	[107]
	DMT1	Acute myocardial infarction and cardiomyocyte hypoxia mouse models	DMT1 knockdown effectively decreased H/R-induced cardiac cell ferroptosis, while overexpression increased it	[108]
Iron storage	Ferritin	Mice lacking ferritin H in cardiomyocytes were feeded these mice a high-iron diet	SLC7A11-mediated ferroptosis causes cardiomyopathy from cardiac ferritin H loss	[109]
Iron transport and utilization	FPN1	Sulforaphane-treated STZ-induced diabetic rats with cardiac IRI models	Activation of NRF2/FPN1 pathway attenuates myocardial ischemia–reperfusion injury in diabetic rats by regulating iron homeostasis and ferroptosis	[65]
		Glucose and hypoxia/reoxygenation-induced cardiomyocytes injury models treated with erastin in vitro		
	Frataxin	Cardiomyocyte-specific HIF-1α knockout mice	Frataxin, an iron storage protein under hypoxia, prevented mitochondrial iron overload and ROS and preserved cardiomyocyte membrane integrity	[110]
Iron homeostasis regulation	Hepcidin	Neonatal mice with apical resection-induced heart regeneration	Macrophages lacking hepcidin promoted cardiomyocyte proliferation	[111]
			IL-6 increased hepcidin in inflammatory macrophages	
	STAT3		Hepcidin deficiency phosphorylated STAT3, releasing IL-4 and IL-13	
	HIF	Cardiomyocyte-specific HIF-1α knockout mice	HIF-1α-frataxin signaling protects against hypoxia/ischemia	[110]

### Cell death

In the type 1 diabetes and type 2 diabetes models, the main forms of diabetic heart cell death include apoptosis, autophagy and necrosis [112, 113]. Tissue homeostasis requires low apoptosis and autophagy to remove unwanted cells, organs, and proteins [27, 112]. However, the increase in apoptosis and the subsequent replacement of fibrosis in the heart is considered a harmful phenomenon [112]. Recently, Cai team [12] confirmed the function of ferroptosis in DC and found that sulforaphane activated NRF2 to prevent DC by suppressing ferroptosis, indicating that DC may be treated by doing so. Because mature mammalian hearts have a limited ability for myocardial regeneration, preventing myocardial cell death may be a key strategy for treating DC [18].

The destiny of cardiac muscle cells is tightly correlated with mitochondrial iron content. The energy required for cardiac physiological activations is provided by iron-sulfur clusters (ISCs), which are produced when mitochondria undergo oxidative phosphorylation [114]. As reported by Wofford et al., iron deficiency may limit energy output, while iron overload may produce excess ROS and lead to mitochondrial destruction [115, 116]. The toxic hydroxyl radicals produced by the reaction of ROS with mitochondria, in addition to the ROS-related toxicity in mitochondria, also contribute to the depolarization of mitochondrial membrane potential (MMP) and the opening of mitochondrial penetration pores, which results in mitochondrial structural swelling and dysfunction [117, 118], and initiating the process of apoptosis and necrosis [119]. At the same time, mitochondrial dysfunction, in turn, leads to mitochondrial iron homeostasis disorders, which further aggravate the ferroptosis.

Mitochondrial iron metabolism is finely regulated by a variety of proteins. Major sources of iron intake from the cytoplasm to the mitochondria are the TF-TFR complex and FT breakdown in lysosomes, which are controlled by mitoferrin 2 (MFRN) and the mitochondrial calcium unit transporter (MCU) [120]. FtMt is a critical regulator of mitochondrial iron homeostasis, particularly in cardiomyocytes, and has a highly homologous sequence to FTH, which has multidirectional effects on iron input by transferring iron from the cytoplasm to the mitochondria [121]. Through this regulatory mechanism, it was found that increased FtMt expression in the heart muscle is the main cause of reduced iron content in mitochondrial LIP, leading to a decrease in systemic ROS production [122]. Studies have demonstrated that the overexpression of FtMt can snare iron from the mitochondria and shield cells against ferroptosis [123]. Therefore, it is conceivable that FtMt could be a potential target for preserving iron homeostasis in cardiomyocytes. Another study revealed that by controlling iron metabolism, preserving mitochondrial function, and elevating GSH levels, the mitochondrial protein iron-sulfur cluster assembase (ISCU) can lessen the toxicity of DHA [79], which is promising as a new target to interfere with ferroptosis.

In fact, a vicious loop that traps cardiomyocytes and worsens ROS-induced damage is created when hypoxic-inducing factor (HIF) is overactive. HIF upregulates the transferrin receptor 1 (TFR1) expression on FtMt [124]. High levels of ROS cause direct oxidative damage to proteins, lipids, and DNA, while ROS is an important trigger for inflammation [125–127]. Studies have shown that inflammation is also one of the pathogenesis of DC patients [128]. Damage-associated molecular patterns (DAMPs) are commonly regarded as immunological mediators for distinct RCD kinds. DAMPs are endogenous molecules that can be generated by dying or dying cells and ultimately induce inflammation and immunological response by attaching to the receptors of different immune cells, such as macrophages and monocytes [129]. Recent studies have shown that HMGB1(high mobility box 1) is a typical DAMP released by ferriogenic cells, activated by macrophages and produced proinflammatory cytokines, causing inflammation [130, 131]. In addition, intermediates or end products of lipid peroxidation may be other sources of modulating immune responses during cell death caused by iron overdose [132]. Due to the minimal immune inflammatory response associated with ferrotosis, specific immunological signals associated with the early or late stages of ferrotosis remain unclear.

### Autophagy

Under normal physiological conditions, the heart’s glucose metabolism is mainly glucose formation, the pathway to pentaphosphate, and the production of glucose. Diabetes alters myocardial substrate utilization [1, 112], resulting in decreased glucose oxidation, increased fatty acid oxidation, and decreased glycolysis. AGE formation and unadapted hexosamine biosynthesis pathways (HBPs) also contribute to glycotoxicity [112, 133]. Diabetes-related cardiac lipotoxicity is not unique to type 2 diabetes hearts, but can also be observed in type 1 diabetes [134, 135]. The utilization of this alteration is due in part to the increased expression of myofilm transporters (especially CD36) on cardiomyocytes that mediate the uptake of free fatty acids to the heart, and the decreased expression and myofilm localization of glucose transporters (i.e. GLUT4) on the heart [112, 134, 136], resulting in increased myocardial lipid content [134, 137]. Both lipotoxicity and glycolipid toxicity can induce autophagy inhibition, and glycolipid toxicity is the most effective inducer of myocardial autophagy inhibition [18]. Because myocardial lipid deposits are associated with high lipid content, diabetes type 1 exacerbates over time [135], it is speculated that chronic glucolipid toxicity induces inhibition of myocardial autophagy in type 1 diabetes. Autophagic defects stop the defense through NRF2, initiate the pathological process of cardiovascular ferroptosis through NRF2, and aggravate DC progression [18]. This suggests that the basic mechanism of DC’s ferroptosis can be linked to NRF2.

NRF2-mediated iron sagging in cardiomyocytes may be downstream of myocardial autophagy inhibition, resulting in the progression of type 1 diabetes cardiomyopathy over time, according to H9C2 cells with impaired autophagy that replicate the cardiac autophagy inhibition phenotype in chronic type 1 diabetes [18]. The unique function of NRF2 in regulating the fate of cardiomyocytes by balancing the expression of genes with opposing roles in cell death is further highlighted by these findings. The underlying dysregulation of NRF2 driver gene expression in many pathological contexts may be the cause of the NRF2-mediated dichotomy. These findings help explain NRF2-mediated cytotoxicity in cardiomyocytes [138], which appears to be a potential inducer of ferroptosis [139].

Ferritin phagocytosis is a mechanism selective autophagy process [140]. Some studies support the idea that activation of ferroptosis depends on induction of autophagy [140–143]. NCOA4 releases more iron from ferritin [85] through ferritin phagocytosis, resulting in ferroptosis. In addition, there is evidence that iron drooping is accelerated when cDNA transfection [141] forces an increase in NCOA4 expression, but is limited when NCOA4 gene is absent [141]. This suggests that excessive expression of NCOA4 genes promotes fertin phagocytosis, releases excess iron, and accelerates cardiac damage through ferroptosis.

### Myocardial fibrosis

The lack of accumulation and structural remodeling of ECM components in the heart (also called myocardial fibrosis) leads to abnormal filling of the left ventricle and diastolic dysfunction [144–146]. Evidence suggests that iron contributes to the development of myocardial fibrosis [147], and the significant presence of myocardial fibrosis is linked to cardiac problems and cardiovascular risk factors [148]. Iron overload may amplify its toxic effect on diabetic heart tissue through oxidative stress, leading to myocardial fibrosis, which is mainly manifested by increased level of type III collagen [149, 150], and aggravates myocardial dysfunction.

It is believed that oxidative damage contributes to iron-mediated cardiac fibroblast activation, resulting in enhanced myocardial fibrosis [148]. An increase in free radical generation from too much iron results in more peroxidation and antioxidant usage [151]. Studies have revealed that oxidative stress affects a pro-fibrosis factor, and that an increase in ROS can stimulate the release of collagen [152, 153]. Calcium channel blockers can prevent iron entrance into cardiomyocytes and lower the volume of collagen in heart tissue since another study revealed that calcium channels are the primary input locations for iron in cardiovascular disorders brought on by iron excess [154–156]. In conclusion, controlling or suppressing the oxidative stress caused by the overload of iron can reduce to some extent the heart fibrosis caused by the overload of iron. At present, the research on the mechanism of myocardial fibrosis mainly focuses on oxidative stress, but research into iron overload in heart failure is very limited and the therapeutic mechanisms of existing drugs are still unknown.

### Microvascular endothelial dysfunction

In the autopsy myocardial samples of diabetic patients, capillaries and arteries decreased and the thickness of the walls of the arteries increased [157]. Both type 1 diabetes and type 2 diabetes patients showed increased coronary resistance and decreased coronary reserve [158], as well as decreased myocardial blood volume and flow [159]. These diabetes-induced microvascular damage reduces the oxygen and other nutrients that are supplied to the heart muscle. These microvascular injuries can further aggravate the impairment of the function of the coronary endothelial cells and the increase in the hardness of the microvascular cells due to persistent diabetes [160–162]. One of the primary target tissues for iron overload injury, which is thought to be caused by an excess of ROS, is vascular endothelial cells (VECs) [163, 164].

In most diabetic patients, serum ferritin levels are high, and ferritin can cause complications in diabetic blood vessels due to iron-induced oxidation stress. High levels of ferritin in type 2 diabetes are closely linked to complications of diabetes vascularity through interactions with VEGF [165]. Endothelial dysfunction is often associated with alterations in the ROS/asymmetric dimethylarginine (ADMA)/eNOS/dimethylarginine dimethylaminohydrolase II (DDAHII) pathway [166]. Too much free iron causes the cytoplasm to overflow with ROS. As a result, excessive ROS activates two vicious cycles—one involving the ADMA/eNOS/DDAHII/NO route and the other involving the ROS-induced ROS release (RIRR) mechanism [167]. The former exerts biological effects in two ways: excessive ROS inhibits DDAHII and accumulates ADMA [167]; ADMA not only competitively inhibits eNOS activity and reduces NO synthesis, but also induces uncoupling of eNOS to produce more ROS, thus making ROS cycle back and back, forming a vicious cycle. The latter, excess ROS enters mitochondria, thereby weakening MMP, opening mPTP, and activating the RIRR mechanism [167, 168], creating another vicious cycle. Together, these two cycles induce a burst of ROS that leads to mitochondrial dysfunction, which in turn damages VECs. Therefore, interrupting any of the steps in the above cycle can end the associated vicious cycle and prevent the onset and progression of injury. For example, quercetin can increase the expression and activity of DDAII through ROS/ADMA/DDAII/eNOS/NO pathway, decrease ADMA level, increase NO content, increase p-eNOS/eNOS ratio, and reduce the oxidative stress and mitochondrial dysfunction induced by iron overload [169].

In addition, recent studies have shown that peroxiredoxin-2 (PRDX2) may play an important pivotal role in ferroptosis-mediated cardiac microvascular injury. PRDX2 is a redox-sensitive thiol-specific peroxidase that protects cells from oxidative stress and is highly expressed in vascular endothelial cells [170]. According to the results of Chen et al. [163], PRDX2 expression is decreased in cardiac microvascular endothelial cells, and endothelium-specific overexpression of PRDX2 can improve mitochondrial function, restore GPX4 expression, reduce Fe^2+^ load and reduce lipid peroxidation accumulation in cardiac microvascular endothelial cells. PRDX2 was identified as a downstream target of Isohesperetin (ISO). ISO, an analogue of resveratrol, can inhibit mitochondrial translocation and mitochondria-related ferroptosis through the PRDX2-MFN2-ACSL4 pathway, and improve the density and perfusion of cardiac microvessels in diabetic patients [163]. These findings open an important new arena for the mechanistic study of the pathogenesis of diabetic cardiomyopathy.

### Ferroptosis in DC-associated cardiomyopathy

In their thorough overview and analysis of the role of ferroptosis in cardiomyopathy, Li et al. [171], underlined that iron fall may one day be used as a treatment for cardiomyopathy. We found that ferroptosis still has many unexplored areas in DC and related cardiomyopathy.

Doxorubicin induces the onset of cardiomyopathy by inserting into mitochondrial DNA and disrupting 5′-aminolevulinic acid synthetase 1-dependent heme synthesis, causing iron ptosis and cardiotoxicity [172]. HCBP6, also known as FUN14 containing domain 2, is a highly conserved and widely expressed mitochondrial outer membrane protein that interacts with the mitochondrial glutathione transporter SLC25A11 to control mitoGSH, which in turn controls iron stress [173]. However, it is still unclear how mitochondria detect stress to initiate ferroptosis under pathological circumstances.

In a recent study, Ferroptosis in hypertensive cardiomyopathy and DC shared 32 differentially expressed genes—26 up-regulated and 6 down-regulated—and three hub genes—periostin, insulin-like growth factor binding protein-5, and fibromodulin [174]. Ferroptosis in DC and hypertension cardiomyopathy is linked to STAT3, lysophosphatidylcholine acyltransferase 3, and solute carrier family 1 member 5. Three hub genes may be cardiomyopathy biomarkers or therapeutic targets [174]. Another bioinformatics analysis found 15 ferroptosis-related genes (4 up-regulated and 11 down-regulated) in ischemic cardiomyopathy and 17 in idiopathic. These genes are mostly engaged in the MAPK signaling pathway in ischemic cardiomyopathy and the PI3K-Akt pathway in idiopathic cardiomyopathy [175]. Future biomarkers for cardiomyopathy prognosis and treatment may include these hub genes and medicines.

## Targeted interventions and prospects

Ferroptosis is mainly characterized by iron overload, lipid peroxidation and the imbalance of antioxidant system. Therefore, in view of the above characteristics, we can consider the following three aspects to inhibit ferroptosis: first, reduce the content of iron in the body, such as dietary restriction of iron intake, promote the utilization of iron ion or accelerate the removal of iron ion; second, lipotoxicity reduction; last, improve the antioxidant capacity, such as the use of antioxidants.

### Drugs or therapies targeting for excess iron

Deferoxamine (DFO), deferasrorox, and deferiprone are FDA-approved cardiac iron chelators, of which DFO decreases mouse heart cell mitochondrial ROS and enhances endothelium-dependent vasodilation in coronary disease patients [176, 177]. Deferasrox is an oral iron chelating agent that protects heart tissue by reducing iron concentration [178, 179]. EDTA(ethylenediamine tetraacetic acid) can alleviate adverse cardiovascular outcomes in patients with acute myocardial infarction [180, 181]. Unfortunately, chelation is complex and has multiple serious adverse effects, such as auditory toxicity, osteotoxicity, and growth retardation [182].

Anti-ferroptosis drugs are considered as a new strategy for the treatment of myocardial injury. Ferrostatin-1, a ferroptosis inhibitor, can alleviate the structural and functional disorders of cardiomyocytes mediated by the loss or low expression of the ferroptosis inhibitor XCT, and reduce the levels of Ang II-induced ferroptosis biomarkers Ptgs2, malondialdehyde and reactive oxygen species [183]. The newly developed polydopamine nanoparticles, as a novel iron ptosis inhibitor, effectively reduced Fe^2+^ deposition and lipid peroxidation in a mouse model of myocardial I/R injury [184] and have interesting properties in restoring mitochondrial function in h9c2 cells. Although this novel technique is still in its infancy, it has great potential for the treatment of cardiac injury caused by ferroptosis.

Canagliflozin, a SGLT2i, can effectively protect the heart in a variety of myocardial injury diseases, such as autoimmune myocarditis [185] and myocardial lipid toxicity [186]. To prevent DC ferroptosis in DC mice and cells, canagliflozin can up-regulate XCT expression, down-regulate ferritin heavy chain expression, and activate the system Xc-/GSH/GPX4 axis [187]. In addition, canagliflozin may significantly inhibit the expression of cyclooxygenase-2 and iNOS by activating AMPK pathway, or inhibit the inflammatory response by reducing the levels of IL-1, IL-6, TNF-α and other inflammatory factors in myocardial cells [188], thereby reducing lipotoxicity and indirectly inhibiting ferroptosis [189]. These studies provide clues for canagliflozin to regulate ferroptosis as an intervention target.

### Drugs or therapies targeting for lipid peroxidation

The introduction of medications to stop the oxidation process is the treatment for ferroptosis, which is brought on by the oxidation of phospholipids. Lipvastatin-1 is a lipophilic RTA (free radical trapping antioxidant) that maintains the DC endothelium cells' ability to function by limiting the spread of free radicals that would otherwise oxidize lipids [190]. These drugs are very effective in the ferroptosis model [191].

However, not all lipid-lowering drugs can reduce iron death by inhibiting lipid peroxidation, such as statins. Fluvastatin, lovastatin, and simvastatin belong to a class of medications called statins that lower blood cholesterol levels by blocking the enzyme HMGCR, which controls cholesterol synthesis in the MVA route. By decreasing the formation of isopentane pyrophosphate in the valvalic acid pathway and limiting the manufacture of selenoproteins (including GPX4) and coenzyme Q10, statins encourage mesenchymal cell iron sags or specifically cause cell death. Therefore, statins may aggravate ferroptosis.

The largest cost on health is posed by the negative consequences of statin-related muscular symptoms (SAMS) [192]. Myocardium is a special striated muscle, and previous reports have shown that the mechanism of myocardial SAMS mainly focuses on mitochondrial dysfunction and apoptosis [193, 194]. For the first time, recent research has demonstrated that atorvastatin produces ferroptosis by blocking the NRF2-XCT/GPX4 pathway of the intracellular antioxidant system, which results in fatal lipid peroxidation in myocytes [195]. In vitro experimental studies, a large amount of Fe^2+^ accumulation, lipid peroxidation and ROS caused by free iron overload induced by atorvastatin through ferroptosis triggered by Fenton reaction in muscle cells, and participated in the process of mitochondrial dysfunction [195]. Therefore, the limitations of statins in clinical application should be fully considered.

In addition, Angiotensin-converting enzyme 2 (ACE2) has been shown to contribute to the reduction of DCM and is a substrate for disintegrin and metalloproteinase protein 17 (ADAM17) [196]. Recent studies have found that the protein expression and activity of ADAM17 are up-regulated in the myocardium of diabetic mice, while the protein expression of ACE2 is down-regulated [24]. The specific knockout of ADAM17 in cardiomyocytes can reduce cardiac fibrosis and cardiomyocyte apoptosis, and improve cardiac dysfunction in DC mice, which may be related to the activation of AMPK pathway, the increase of autophagosome formation and the improvement of autophagic flux [24], and indirectly regulate the process of lipid metabolism. These results suggest that ADAM17 inhibition may provide another promising approach for the treatment of DC.

### Targeted enhancement of antioxidant

NRF2 is recognized as an important antioxidant defence regulator and is developed as a promising target drug for DC treatment [197]. In recent years, the two most promising approaches to limiting the damage of the oxidative heart caused by diabetes are to inhibit NADPH oxidation by pharmaceutical activation of NRF2 [126, 198, 199] and combined NOX1/NOX4 inhibitor GKT137831, as demonstrated in several preclinical models of diabetes [200–202].

The current research on NRF2 in the treatment of ferroptosis is mainly focused on myocardial ischemia–reperfusion, and there is a lack of research on DC. Dexmedetomidine can inhibit ferroptosis through AMPK/GSK-3β/NRF2 axis [203], or by regulating the expression of ferroptosis-related proteins [204], including SLC7A11, GPX4, ferritin heavy chain and cyclooxygenase-2, and activating SLC7A11/GPX4 axis. Reduce myocardial ischemia–reperfusion injury in rats.

In addition, many traditional Chinese medicine monomers and preparations have shown good effects on inhibiting ferroptosis by targeting NRF2-related pathway. Berberine hydrochloride increased cell viability and MMP by inhibiting NRF2 [205] or by reducing ROS generation and lipid peroxidation [206], showing a good inhibitory effect on ferroptosis. Britanin can alleviate ferroptosis by activating AMPK/GSK3β/NRF2 signaling pathway and up-regulating GPX4 [207]. Naringenin [23] and Shenmai injection [208] can inhibit iron ptosis by regulating NRF2/System XC-/GPX4 axis. 6-Gingerol [209] and curcumin [210] can alleviate myocardial injury in DC mice and cell models and activate the NRF2/HO-1 pathway by enhancing GPX4 expression. In addition, 6-Gingerol can also reduce the secretion of inflammatory factors IL-1β, IL-6 and TNF-α [209] to alleviate the inflammatory response.

However, there is considerable controversy in the current literature on the role and complications of NRF2 in diabetes. For example, global low phenotypic knockout of the endogenous inhibitor of NRF2, Kelch-like ECH-associated protein 1 (Keap1), activates the NRF2 gene and thus inhibits the development of diabetes in db/db mice [211]. However, paradoxically, it increased insulin resistance and glucose intolerance in mice [212]. The exact reason for these differences is unknown. Clinical trials on various stages of NRF2 activators in the treatment of other diseases are still being conducted [213].

The development of drugs that target the gut microbiota and targeted therapy, in addition to NRF2 and related pathways, has opened up a new realm for the treatment of ferroptosis. Salidroside is the main component of traditional Chinese medicine Rhodiola. Salidroside can increase the proportion of probiotics and reduce the proportion of lactic acid bacteria [214], among which iron metabolism is related to the abundance of lactic acid bacteria, thereby reshaping the intestinal flora and limiting the accumulation of iron. Additionally, by activating the AMPK-dependent signaling pathway, salidroside can restore mitochondrial membrane potential, improve mitochondrial biogenesis, enhance mitochondrial iron-sulfur clusters, restore mitochondrial OXPHOS complex, inhibit DOX-induced mitochondrial ROS, Fe^2+^, and lipid peroxidation, and restore mitochondrial ROS and Fe^2+^, these effects enhance mitochondrial function while defending cardiomyocytes [215].

Recent research has shown that 2-vinyl-10H-phenothiazine derivatives, which play the role of ROS scavengers and can treat DOX-induced cardiomyopathy, can be employed as a family of ferroptosis inhibitors with limited human Ether-a-go-go associated gene activity [216]. While, it shows good pharmacokinetic properties and no obvious toxicity in vitro and in vivo, which provides a promising lead compound for the development of drugs targeting ferroptosis.

## Conclusions

Different from other types of cell death, ferroptosis is a new type of cell death that depends on iron overload and lipid peroxidation. Increasing evidence suggests that ferroptosis is widely involved in the occurrence and development of DC. Cardiomyocytes and microvascular endothelial cells are the main sites of ferroptosis in DC, involving multiple different pathological pathways such as mitochondrial dysfunction, oxidative stress, immune inflammatory response and so on. Among them, oxidative stress runs through the whole process of the disease.

Improving lipid metabolism, improving antioxidant capacity and promoting iron metabolism are essential for the treatment of DC. According to existing studies, ferroptosis and disease progression can be effectively inhibited by reducing excess iron, enhancing the antioxidant capacity of the body and inhibiting inflammation. For example, iron inhibitors, iron chelators, some lipid-lowering drugs and antioxidant drugs have shown good effects on inhibiting ferroptosis and protecting cardiomyocytes in vitro and in vivo. Bioinformatics has shown unique advantages in screening key genes and core proteins, which provides guidance for targeted intervention. Meanwhile, the application of some emerging technologies has also created a precedent for the removal of excessive ROS. The development of nanoparticle technology provides a new prospect for exploring new drug carriers. These will prompt us to search for new alternative drugs or new drug carrier technologies.

Currently, ferroptosis research in cardiovascular diseases is limited and focuses mainly on animal and in vitro experiments. There are not enough clinical trials and observations on human cardiovascular diseases. Therefore, the specific pathogenic pathways and mechanisms of ferroptosis in the huge and complex regulatory system of human body remain unclear. However, it is undeniable that the animal and cell experiments of ferroptosis in DC have laid a solid foundation for the study of DC and its related cardiomyopathy, connecting the treatment of cancer and cardiovascular diseases through the application of iron chelators, which opens up a new perspective for the mechanism research of DC and the joint research of different disciplines.

## Data Availability

Not applicable.
